# Translabyrinthine resection of NF2 associated vestibular schwannoma with cochlear implant insertion

**DOI:** 10.3171/2021.7.FOCVID21122

**Published:** 2021-10-01

**Authors:** Cathal John Hannan, Priya Sharma, Matthew Edward Smith, Laurence Johann Glancz, Martin O’Driscoll, Andrew Thomas King, Charlotte Hammerbeck-Ward, Dafydd Gareth Evans, Scott Alexander Rutherford, Simon Kinsella Lloyd, Simon Richard Mackenzie Freeman, Omar Nathan Pathmanaban

**Affiliations:** 1 Department of Neurosurgery, Manchester Centre for Clinical Neurosciences, Manchester;; 2 Geoffrey Jefferson Brain Research Centre, Manchester;; 3 Faculty of Medicine, Imperial College London;; 4 Department of Otolaryngology, Salford Royal NHS Foundation Trust, Manchester;; Departments of 5 Otolaryngology and; 6 Audiology, Manchester University NHS Foundation Trust, Manchester;; 7 Division of Cardiovascular Sciences, School of Medical Sciences, Faculty of Biology, Medicine and Health, University of Manchester;; 8 Manchester Centre for Genomic Medicine, St. Mary’s Hospital, Manchester University NHS Foundation Trust, Manchester;; 9 Division of Evolution and Genomic Sciences, School of Biological Sciences, Faculty of Biology, Medicine and Health, University of Manchester, Manchester Academic Health Science Centre, Manchester; and; 10 Division of Neuroscience and Experimental Psychology, School of Biological Sciences, Faculty of Biology, Medicine and Health, University of Manchester, United Kingdom

**Keywords:** vestibular schwannoma, neurofibromatosis type 2, hearing rehabilitation

## Abstract

The authors present the case of a 24-year-old female with neurofibromatosis type 2. Growth of the left vestibular schwannoma and progressive hearing loss prompted the decision to proceed to translabyrinthine resection with cochlear nerve preservation and cochlear implant insertion. Complete resection with preservation of the facial and cochlear nerves was achieved. The patient had grade 1 facial function and was discharged on postoperative day 4 following suturing of a minor CSF leak. This case highlights the feasibility of cochlear nerve preservation and cochlear implant insertion in appropriately selected patients, offering a combination of effective tumor control and hearing rehabilitation.

The video can be found here: https://stream.cadmore.media/r10.3171/2021.7.FOCVID21122

## Transcript

In this video, we present the case of a translabyrinthine resection of a neurofibromatosis type 2 (NF2)–associated vestibular schwannoma (VS) with simultaneous cochlear implant insertion.

### 0:30 Case History

The patient is a 24-year-old medical student, managed through the supraregional NF2 service based in Manchester. She was diagnosed with bilateral VS, managed initially with radiological observation. However, slowly progressive growth was demonstrated on recent surveillance imaging, more so of the left-sided tumor. Intracranial imaging from 2020 demonstrated an increase in maximal linear diameter of the tumor of around 2 mm, when compared to imaging from 1 year previously. There was also progressive hearing loss in the left ear.

### 1:02 Preoperative Imaging

Postcontrast T1-weighted MRI demonstrated the presence of bilateral vestibular schwannomata, larger on the left side, as previously described. There was evidence of tumor extension laterally to the fundus of the IAC and into the vestibule.

There were no intracranial meningiomas or ependymomas.

### 1:20 Preoperative Audiometry

Successive audiometry demonstrated a progressive deterioration in pure tone average and speech discrimination score in the left ear. The patient’s most recent pure tone audiogram is displayed on the slide, demonstrating severe to profound sensorineural hearing loss in the left ear. The patient had previously trialed a hearing aid in this ear, and not found it to be helpful, due to amplification of background noise and the inability to focus on individual conversations due to distortion, a problem commonly encountered with the use of hearing aids in NF2.^1^

Hearing in the right ear was normal.

### 1:53 Discussion of Management Options

Given the degree of preoperative hearing loss, the patient was not a candidate for hearing preservation surgery.

Although stereotactic radiosurgery was considered, we elected against this approach due to the poorer tumor control in NF2-associated vestibular schwannomata as compared to sporadic tumors, the small but nonetheless present risk of malignant transformation of the VS in a patient with a tumor predisposition syndrome, and the suggestion in the literature of poorer hearing outcomes associated with CI implantation in the setting of an irradiated VS.^2–4^

The decision to proceed with a translabyrinthine approach and resection of the tumor, with simultaneous cochlear implant insertion, was taken for a number of reasons.

Firstly, the progressive growth of the tumor and loss of hearing in the ipsilateral ear justified an active approach, rather than continued conservative management. Moreover, the tumor was of a size whereby preservation of facial nerve function was highly likely, which was an absolute priority for the patient.^5^ Due to the lateral extension of the tumor into the vestibule, the translabyrinthine approach was preferred as we felt this provided the best chance of achieving a gross-total resection and minimizing the requirement for further interventions in future.

Given the presence of a contralateral VS, effective hearing rehabilitation in the affected ear would be even more valuable in the event of deterioration in hearing on the contralateral side. Finally, the patient had recently graduated from medical school, and the hiatus between graduation and commencing work as a doctor offered an opportunity to perform the surgery at a time when it was likely to be minimally disruptive for the patient, both personally and professionally.

Cochlear nerve–preserving surgery with concomitant cochlear implant insertion is an approach supported by our own institutional experience, as well as by recent systematic reviews demonstrating positive results with cochlear implantation in the setting of NF2, particularly when compared to auditory brainstem implantation.^2,6–8^

### 3:44 Patient Positioning and Initial Skin Incision

Following the induction of general anesthesia, the patient was positioned supine with the head turned to the right in a gel head ring. A curved retroauricular incision was planned. Electrodes for facial nerve EMG, facial MEP, and electronically evoked auditory brainstem response (eABR) were placed. Given that we wished to monitor facial motor evoked potentials and continuous facial EMG, inhaled anesthetic agents and muscle relaxants were avoided by our anesthesiology team.

Following initial skin incision, a myocutaneous flap was raised anteriorly and secured with fishhooks to expose the mastoid bone.

### 4:11 Mastoidectomy/Labryinthectomy

With the mastoid exposed, a cortical mastoidectomy was performed in standard fashion, with skeletonization of the sigmoid sinus, exposure of middle fossa dura, and delineation of the fallopian canal.

Exposed dura was cauterized, prior to posterior mobilization of the sigmoid sinus and bony labyrinthectomy. Following entry into the vestibule, tumor tissue was encountered and removed.

### 5:09 Exposure of Internal Auditory Canal Dura

Gutters were drilled at the superior and inferior aspects of the internal auditory canal, with the aim of exposing it through 270°. Following this, the bone overlying the dura of the internal auditory canal was elevated and gently removed.

### 5:29 Removal of Incus/Placement of eABR Electrode

After removal of the bone surrounding the internal auditory canal, the incus was removed and the epitympanotomy window widened, to allow access to the round window for the placement of the golf club eABR electrode.

It was not possible to record eABR waveforms at this juncture, due to interference from stimulation of the facial nerve.

### 5:59 Dural Opening/Identification of Surrounding Structures

The dura overlying the tumor was opened with scissors, and the dural edges tacked out of the operative field with stay sutures. Arachnoid webs inferior to the tumor were divided to release CSF.

A 45° endoscope was introduced, providing clear views of the facial and vestibulocochlear nerves as they exited the brainstem, as well as an intervening loop of AICA and the schwannoma arising more laterally.

Endoscopic views were also obtained of the dorsal cochlear nucleus; however, attempts at eABR recording using a ball electrode were once again impeded by interference from facial nerve stimulation.

### 6:48 Tumor Resection

Tumor dissection was initiated with the establishment of a plane between the tumor and the cochlear nerve, using microdissectors. The CP angle component of the tumor was partially debulked, before moving laterally to the internal auditory canal, where further drilling of the fundus of the internal auditory canal exposed the most lateral aspect of the tumor.

The tumor was meticulously dissected from lateral to medial, with avulsion of the superior vestibular nerve to facilitate exposure of the underlying facial nerve.

Final stages of the resection involved the removal of tumor components adherent to the cochlear nerve and resection of the CP angle component. Due to the lack of a clear plane between the lateral aspect of the facial nerve and the tumor, the final stages of tumor resection were facilitated by sharp dissection. A tiny volume of tumor residuum was left on the facial nerve, as we felt further attempts at establishing a plane between this very small aggregation of tumor cells and the nerve would compromise postoperative facial function.

### 8:00 Final Overview

Final overview confirms near-total resection, with anatomical preservation of the facial and cochlear nerves. Also visible is the preserved loop of AICA, which was interposed between the tumor and the cochlear and facial nerves, as well as lying in between the cochlear and facial nerves.

The facial nerve was stimulating briskly at 0.05 mA at the conclusion of the tumor resection.

### 8:23 Cochlear Implant Insertion/Closure

Following tumor resection, the cochlear implant was inserted. Given that facial nerve monitoring was no longer required, short-acting muscle relaxants were administered by our anesthesiologists to allow for accurate recording of eABRs.

The eustachian tube and middle ear were obliterated with muscle, and abdominal fat was packed into the petrosectomy defect and secured with a dural sealant. The scalp was closed in layers with absorbable sutures to the skin.

eABR recordings were successfully recorded while stimulating using the implant, as demonstrated on the recordings obtained by the audiologist.

### 8:57 Postoperative Course

The patient emerged from anesthesia with no neurological deficits and House-Brackmann grade I facial function. The patient recovered well and was discharged on postoperative day 4 following suturing of a minor CSF leak on the floor. She experienced no vestibular symptoms postoperatively, which we attribute at least partially to our administration of intratympanic gentamicin preoperatively, which has been instrumental in reducing our length of stay following VS surgery.^9^

The patient will continue to be reviewed under the auspices of the NF2 service in Manchester, with a plan for continued radiological surveillance of the contralateral VS.

### 9:32 Cochlear Implant Programming

The patient returned for programming of her cochlear implant 6 weeks postoperatively and was able to hear on stimulation of all 22 cochlear implant electrodes, with no nonauditory side effects. The patient will return for ongoing CI programming and audiometric follow-up.

### 9:49 Conclusion

It is clear that the clinical decision-making with respect to the management of NF2-associated vestibular schwannomata is invariably complex.

However, this case serves to illustrate that in judiciously selected patients, resection of their VS with cochlear nerve preservation and cochlear implant insertion allows for definitive treatment of the tumor alongside hearing rehabilitation, while offering a high chance of facial nerve preservation in tumors of the appropriate size.

## Disclosures

Dr. Evans reports personal fees from AstraZeneca, outside the submitted work.

## Author Contributions

Primary surgeon: Pathmanaban, Freeman. Assistant surgeon: Smith, Glancz, Hammerbeck-Ward, Rutherford. Editing and drafting the video and abstract: Pathmanaban, Hannan, Smith, Glancz, Hammerbeck-Ward, Evans, Lloyd, Freeman. Critically revising the work: Pathmanaban, Hannan, Sharma, Smith, Glancz, O’Driscoll, King, Evans, Rutherford, Lloyd, Freeman. Reviewed submitted version of the work: Pathmanaban, Hannan, Sharma, Hammerbeck-Ward, Evans, Rutherford, Lloyd, Freeman. Approved the final version of the work on behalf of all authors: Pathmanaban. Supervision: Pathmanaban, King.
